# Ovine Tendon Collagen as Dermal Substitute for Hand Wound Coverage: A Case Report

**DOI:** 10.5704/MOJ.2511.018

**Published:** 2025-11

**Authors:** MS Azhar, S Abdullah, AYW Poh, EZF Soh, CH Lim

**Affiliations:** 1 Department of Orthopaedics and Traumatology, Universiti Kebangsaan Malaysia, Kuala Lumpur, Malaysia; 2 Unit of Plastic and Reconstructive Surgery, Universiti Kebangsaan Malaysia, Kuala Lumpur, Malaysia

**Keywords:** Ovine tendon collagen, hand-wound, wound coverage

## Abstract

Deep-space infection of the hand can be associated with a varying degree of morbidity. Management will always start with an incision and drainage of the affected hand, followed by appropriate intravenous antibiotic therapy and mobilisation of the joint post-operatively. In order to achieve a good outcome, wound bed preparation is vital as the first stage of wound healing. Ovine Tendon Collagen (OTC) can be a new alternative source of collagen that can be used as a bio scaffold for skin defect coverage.

## Introduction

Wound coverage following a radical incision and drainage of a deep-space infection has always been a challenge. Devitalised tissue and contaminated wound beds warrant multiple wound debridement after the initial operation. In view of extensive tissue loss and ongoing infection, wounds are usually left open either for later closure or secondary healing. In such cases, Ovine Tendon Collagen (OTC) presents a valuable option for wound bed preparation1.

Ovine tendon collagen (OTC), extracted from the connective tissues of sheep, stands out for its unique properties, including its high biocompatibility and similarity to human collagen, making it an ideal candidate for biomedical applications such as wound healing. Its superior tensile strength and durability make it particularly suitable for enhancing the mechanical properties of biomaterials.

In this study, we will be focusing on how OTC can be used as a dermal substitute for wound coverage in hand wounds.

## Case Report

A 65-year-old female presented to our emergency department with a one-week history of right-hand swelling associated with pus discharge following a shrimp prick. On examination, the right-hand hypothenar area appeared swollen, extending to the midpalmar area. The affected area was fluctuant in consistency, with the punctum located over the lateral aspect of the distal palmar crease. Blood investigations showed elevated white blood cell and C-reactive protein levels. Radiological imaging showed no evidence of osteomyelitis or retained foreign bodies.

**Fig. 1:**
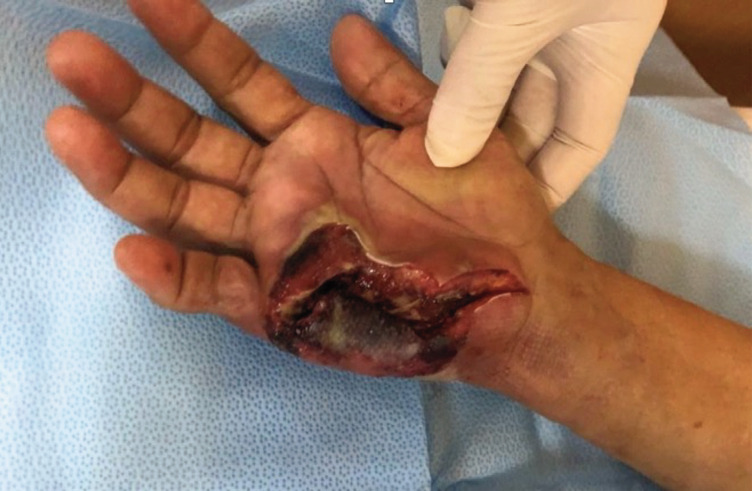
Day one post-operative clinical picture showing flap necrosis with a sloughy wound bed.

An emergency incision and drainage was performed, draining 5cc of purulent material. The wound was left open and packed with Hydrocyn Aqua and Bactigras dressing. However, during the next-day wound inspection, we faced a common complication of hand surgery, which was flap necrosis thus leaving the wound with a significant area of tissue loss (Fig. 1). The patient was counselled for OTC application and consented to the intervention. On Day 3 post-operatively the wound appeared clean and free from infection, allowing for the subsequent application of OTC.

**Fig. 2:**
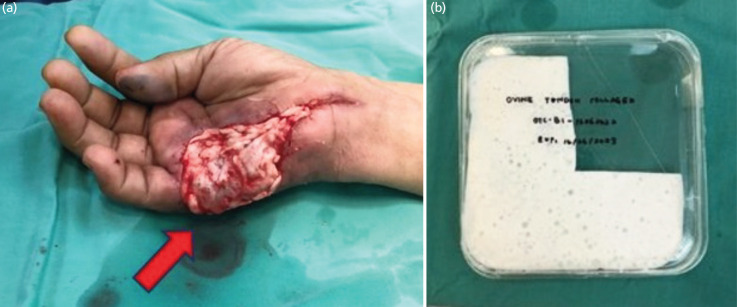
(a) Showing OTC application for the dermal substitute of right hand wound. (b) Showing OTC in a mesh form.

**Fig. 3:**
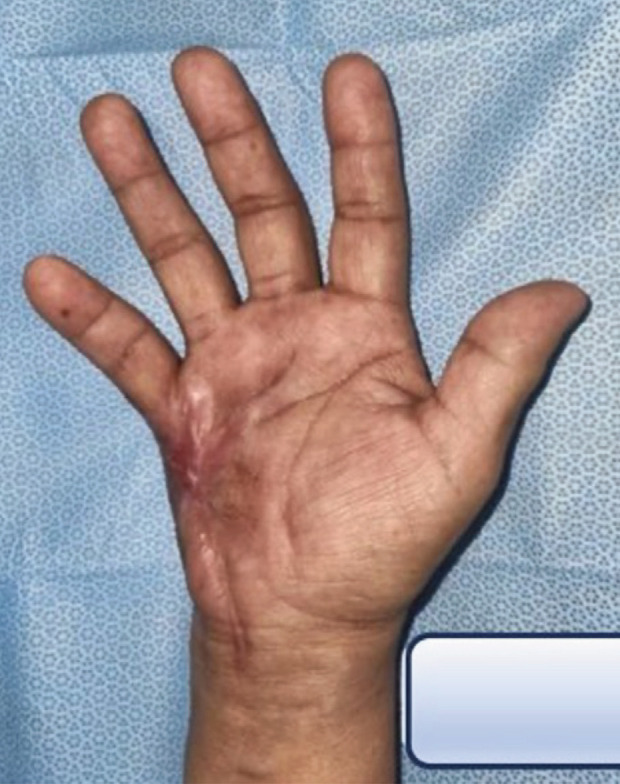
Post-operative clinical picture of the right hand.

OTC was provided by the Hospital Canselor Tuanku Muhriz (HCTM) plastic team, and the application was performed in the operation theatre. A 5×5cm sheet of OTC was positioned over the wound and sutured to the wound edges using Monosyn 3/0 (Fig. 2) and dressed with bactigras and gauze on top. Daily dressing changes were maintained for three weeks post-operatively until complete wound closure was achieved (Fig. 3).

## Discussion

OTC is a new alternative option for the establishment of the dermis in cases of full-thickness wounds. Mh Busra *et al* highlighted the ability of OTC to facilitate the regeneration of skin tissue, providing a supportive matrix for cellular ingrowth and promoting tissue repair^1^. Similarly, another study compared the attachment, proliferation, and morphological properties of human dermal fibroblasts on OTC scaffolds, emphasising its suitability as a substrate for cell growth and tissue integration^2-3^.

The concept behind OTC application is to efficiently reconstruct the dermis by facilitating the migration of fibroblasts, macrophages, lymphocytes, and capillary ingrowth onto its surface^4^. In order for the OTC to work, the wound needs to be debrided properly, and it is important to ensure that the wound bed is free from any contamination or infection. OTC will be applied above the dermal layer of the wound, and clinical improvements can be observed as early as seven days post-application.

For this patient, OTC was applied after wound debridement. The wound was thoroughly debrided and washed with copious amounts of saline before the application. The 5x5cm OTC was supplied by the plastic team HCTM. and it was applied using absorbable suture Monosyn 3/0 to the dermal layer. A protective dressing consisting of gauze impregnated with paraffin and chlorhexidine was placed over the OTC to minimise traumatic removal and reduce infection risk. Finally, another layer of dry gauze was applied to the wound, and light bandaging was applied. The wound was left undisturbed for five days to facilitate the initial phase of healing prior to the next inspection.

Upon inspection, granulation tissue can be seen from the wound bed with good uptake as well as a reduction in the wound size. OTC is proven to speed up the wound healing process by increasing the rate of epithelial cell proliferation and differentiation from fibroblasts to myofibroblasts. These two factors are important components in contracting the granulation tissue and drawing the wound edges closer.

One of the key advantages of ovine tendon collagen lies in its structural similarity to human collagen, significantly reducing the risk of immunological rejection or adverse tissue reactions. Based on the previous study, OTC is proven to have low immunogenicity and has remarkable biocompatibility with the recipient site^5^. This was evidenced in the current case, where good uptake was observed without any complications throughout the treatment period. There was no need for wound debridement following the application, and by the end of two weeks, the wound appeared smaller in size with healthy epithelialization tissue.

There are multiple options of bovine and porcine dermal substitutes but their use is restricted in Hindu and Muslim communities due to religious prohibitions. Another further key advantage of an ovine dermal substitute would be its suitability in a multi-religious country or a global, multi-religious setting.

In order to reduce scar contracture and facilitate wound healing, split-thickness skin grafting was advised to the patient. However, due to personal reasons, she refused the procedure, so daily dressings with Hydrocyn and Bactigras was applied to the patient. This conservative approach still resulted in complete wound closure within three weeks.

In conclusion, the treatment of a hand abscess can be a challenging process. Proper drainage is vital in the early stages of the treatment, followed by wound bed preparation later on. Overall, the use of ovine tendon collagen as a dermal substitute offers a promising approach to wound management, providing an effective, safe, and biocompatible solution for promoting healing and restoring skin integrity. Continued research and development in this field could further enhance its efficacy and expand its clinical applications.
